# Investigating retrotransposon‐derived DNA and activation of the innate immune response in tauopathy

**DOI:** 10.1002/alz70861_108015

**Published:** 2025-12-23

**Authors:** Morgan Elizabeth Lambert, Paulino Ramirez, Wenyan Sun, Bess Frost

**Affiliations:** ^1^ Robert J. and Nancy D. Carney Institute for Brain Science, Providence, RI USA; ^2^ Center for Alzheimer's Disease Research, Providence, RI USA; ^3^ Brown University, Providence, RI USA; ^4^ University of Missouri‐Kansas City, Kansas City, MO USA

## Abstract

**Background:**

Extrachromosomal circular DNA (eccDNA) was extracted from post‐mortem human brain with tauopathy and sequenced. Cytosolic DNA binding proteins and their RNA transcripts were quantified in induced pluripotent stem cells (iPSCs) with tauopathy.

**Method:**

DNA was extracted from frontal cortex of Alzheimer's disease Braak III and Braak VI, progressive supranuclear palsy, and healthy control post‐mortem brains (n = 6). Linear DNA was digested with exonuclease V and VIII, then purified to isolate eccDNA. The eccDNA and endogenous genomic DNA per patient were sequenced. RNA was extracted from iPSC‐derived neurons harboring a pathogenic tau mutation as well as isogenic controls and sequenced (n = 7). Protein was extracted and quantified.

**Result:**

Braak III eccDNA was enriched for retrotransposon DNA. Cytosolic DNA sensor and inflammasome component AIM2 was upregulated at the RNA transcript level in iPSC‐derived neurons with tauopathy. Innate immune protein STING1 was downregulated in iPSC‐derived neurons with tauopathy.

**Conclusion:**

Retrotransposons may be forming eccDNA and potentially activating cytosolic DNA‐sensing proteins in tauopathy.

